# Incidence and impact on clinical outcome of infections with piperacillin/tazobactam resistant *Escherichia coli *in ICU: A retrospective study

**DOI:** 10.1186/1471-2334-8-67

**Published:** 2008-05-17

**Authors:** Agnès Meybeck, Jean-Damien Ricard, Guilène Barnaud, Mathieu Eveillard, Guillaume Chevrel, Roman Mounier, Didier Dreyfuss

**Affiliations:** 1 Service de Réanimation, Hôpital Louis-Mourier, 178 rue des Renouillers 92701 Colombes Cedex, France; 2 Service de Bacteriologie, Hôpital Louis-Mourier, 178 rue des Renouillers 92701 Colombes Cedex, France

## Abstract

**Background:**

*Escherichia coli *infections are frequent in ICU patients. The increased resistance to fluoroquinolones and amoxicillin/clavulanate of this pathogen mandates the prescription of broad-spectrum antibiotics such as piperacillin/tazobactam (PIP-TAZ) or third generation cephalosporins (3GC).

**Methods:**

To assess incidence and impact on clinical outcome of infections with PIP-TAZ resistant *E. coli *in ICU patients, we conducted a retrospective cohort study with infections due to PIP-TAZ resistant (PIP-TAZ R) or to PIP-TAZ susceptible strains (PIP-TAZ S) between 1 January 2002 and 30 June 2004.

**Results:**

Of 83 strains, 13 were PIP-TAZ R: 2 strains produced an extended-spectrum β-lactamase (2%), 11 produced a high level penicillinase (13%). Prior amoxicillin or amoxicillin/clavulanate prescription was reported in 7 cases (54%) of infections with PIP-TAZ R isolates and in 15 cases (21%) of infections with PIP-TAZ S isolates (p = 0.03). Time of onset of the infection from hospital admission was longer in case of infections with PIP-TAZ R than with PIP-TAZ S isolates (22 ± 32 vs 10 ± 21 days, p = 0.01). The overall ICU mortality rate was 38%. Mortality and length of stay in ICU were similar in case of infections with PIP-TAZ R isolates and with PIP-TAZ S isolates.

**Conclusion:**

Infections with PIP-TAZ R *E. coli *are frequent in ICU patients. No prognostic impact of this pattern of resistance was found. Prescription of PIP-TAZ for empirical treatment of *E. coli *infections in ICU however exposes to inappropriate therapy.

## Background

The development of resistance to antimicrobial agents has been an ongoing and evolving process since antibiotics were introduced a half-century ago. Antimicrobial-resistant pathogens are becoming a prevalent cause of hospital-acquired infections, particularly in intensive care units (ICU) [1]. Members of the family Enterobacteriaceae are the most frequent organisms isolated in clinical microbiological laboratories. *Escherichia coli *is responsible of both community and nosocomial infection. It is frequently involved in sepsis in critically ill patients [2]. It is becoming increasingly resistant to commonly used antibiotics such as fluoroquinolones or amoxicillin-clavulanic acid [3]. The widespread presence of resistant bacteria in ICU has led to the common practice of using broad-spectrum antimicrobial coverage in case of suspected gram-negative sepsis. Piperacillin/tazobactam is an injectable antibiotic mixture consisting of the semisynthetic antibiotic piperacillin-sodium (PIP) and the betalactamase inhibitor tazobactam-sodium (TAZ). PIP-TAZ antimicrobial spectrum includes both Gram-positive cocci and Gram-negative bacilli, especially enterobacteriaceae and *Pseudomonas aeruginosa*. Clinical use of PIP-TAZ is wide, especially in ICU. It is notably proposed for the treatment of severe intra-abdominal infections, febrile neutropenic patients at high risk, late-onset ventilator-associated pneumonia [4-6]. Recent reports pointed out the increase in the prevalence of PIP-TAZ resistant strains of *E. coli *isolated from clinical relevant specimens in ICU [7,8]. Data regarding the impact of antimicrobial-resistant pathogens on clinical outcome demonstrated deleterious effects with higher length of stay and mortality rate [9,10]. This impact was demonstrated in case of infections due to various pathogens including *E. coli*. Previous studies focusing on the impact of *E. coli *resistance concerned essentially ESBL-producing strains and more recently resistance to fluoroquinolones [11,12]. We conducted a retrospective cohort study to specially investigate the incidence of PIP-TAZ resistance and its impact on clinical outcome of infections with *E. coli *in critically ill patients.

## Methods

### Patients

The study was conducted at the Louis Mourier Teaching Hospital ICU (Colombes, France), a 450-bed tertiary care university hospital. The ICU is a 14-bed medical-surgical unit. The investigation was conducted over a period of 2.5 years (1 January 2002 through 30 June 2004).

All clinical cultures demonstrating an *E. coli *isolate were identified through records of the clinical microbiology laboratory of Louis Mourier Teaching Hospital. Identification and susceptibility testing of *E. coli *isolates were performed by standard methods, using the API system 20E (Bio Mérieux^® ^SA, Marcy l'Etoile, France). The resistance phenotype was determined by the disk diffusion method with Mueller-Hinton agar [13]. Results were interpreted in accordance with the recommendations of the Antibiogram Committee of the French Society for Microbiology [14]. An isolate was considered to be intermediate if it demonstrated a PIP-TAZ MIC ≥ 8 mg/L and resistant if MIC was > 64 mg/L. For the purpose of this study, intermediate and resistant to PIP-TAZ *E. coli *strains were grouped together under the denomination of PIP-TAZ R strains. Resistance to cefotaxime was used as a marker for resistance to third generation cephalosporins (3GC). An isolate was considered to be resistant if it demonstrated a cefotaxime MIC ≥ 4 mg/L. We identified the strains resistant to both PIP-TAZ and third generation cephalosporins (*E. coli *PIP-TAZ R/3GC R) and the strains resistant only to PIP-TAZ (*E. coli *PIP-TAZ R/3GC S).

After the selection of patients with *E. coli *isolates, medical records of these patients were reviewed to determine whether each patient met inclusion criteria. Only patients whose isolates were responsible for infection (as opposed to colonization) were included. Isolates from the same patient with the same pattern identified in the course of the same infectious episode were excluded. Infection was defined as the presence of *E. coli *in a sterile milieu (such as blood, cerebral fluid, pleural fluid, or ascitic fluid) and/or clinically suspected infection (fever or hypothermia, leukocytosis, signs of focal infection), and the identification of *E. coli *in significant quantity from a clinical sample.

Infection was considered hospital-acquired if it appeared 48 hours after hospital admission and no evidence of infection was present on admission. All other infections were considered community acquired.

### Data collection

Data were collected through review of inpatient medical records. Data obtained included age, sex, race, the indication(s) for ICU admission, underlying clinical conditions, presence of immunosuppression, severity of illness at ICU admission. The underlying clinical conditions were classified according to the criteria proposed by McCabe and Jackson in 3 categories: non fatal, ultimately fatal and rapidly fatal [15]. Immunosuppression was defined as a leukocyte count less than 1000/mm^3^, recent use of systemic corticosteroids (more than 10 mg/d of prednisone or equivalent for over 2 weeks), underlying malignancy, cytotoxic drugs, radiation treatment, or asplenia. The severity of illness at the time of ICU admission was assessed by SAPS II and Acute Organ System Failure (OSF) scoring system [16,17]. Neurological and mental status was stratified according to Glasgow Coma Score [18]. All antibiotics instituted within 1 month prior to the occurrence of infection were taken into account.

At the time of infection onset, the following data were recorded: the number of hospital days before infection, the number of ICU days before infection, the site of infection, the presence of bloodstream involvement, the presence of a coinfecting organism.

The following features of the treatment for each infection were documented: the initial antimicrobial therapy, the time between infection onset and initiation of adequate antimicrobial therapy (defined as the prescription of at least one molecule to which *E. coli *was susceptible). Our primary outcome of interest was mortality in ICU. Secondary outcomes of interest were the length of stay in ICU, the occurrence of sepsis-related complications (secondary septic shock, ARDS, development of MOF, superinfection, infection relapse). Multiple organ failure (MOF) was defined as the dysfunction of two or more of the six evaluated organ systems accorded to Fagon et al. definitions [19]. Acute respiratory distress syndrome (ARDS) was defined according to usual criteria [20].

### Statistical methods

Categorical variables were compared using Chi-square test or Fisher's exact test when Chi-square was not appropriate. Continuous variables were compared using Student's t test. Differences between groups were considered to be significant for variables yielding a p value ≤ 0.05.

## Results

### Demographics

A total of 83 *E. coli *strains responsible for an infection were isolated during the study period. The strain was PIP-TAZ R/3GC R in two cases. Both strains produced extended-spectrum β-lactamase. In these two cases the infection was acquired in the ICU. In the other 81 cases of *E. coli *infection, the strain was sensitive to third generation cephalosporins, and resistant to PIP-TAZ (PIP-TAZ R/3GC S) in 11/81 cases. In these 11 cases, the strain produced a high-level penicillinase.

The demographic data at ICU admission for the 83 patients infected with a *E. coli *strain are shown in Table 1. The mean age was 61 ± 16 years and 51% were male. Medical conditions accounted for 96% of total admissions. Infections accounted for 36% of admissions, respiratory failures for 22%, and neurologic diagnoses for 20%. No significant differences where observed between patients infected with PIP-TAZ R isolates and with PIP-TAZ S isolates with regard to age, gender, SAPS II, Glasgow scores, immune status, and presence of an underlying fatal medical condition. OSF score was significantly higher in case of infection with PIP-TAZ R strains.

**Table 1:** Patient characteristics on ICU admission *.

**Characteristics**	**PIP-TAZ R **n = 13	**PIP-TAZ S **n = 70
Age (years)	61 ± 15	61 ± 16
Gender (Male/Female)	8/5	34/36
*Indications for ICU admission*		
Infectious diseases	7	23
Respiratory failure	4	13
Neurological disturbances	0	17
Shock	1	6
Metabolic disturbances	1	5
Surgical diagnosis	0	3
Others	0	4
*Underlying diseases*		
Anticipated death within 5 years **‡**	8 (61)	33 (47)
Immunosuppression	7 (54)	24 (34)
*Severity of illnesses*		
SAPS II	63 ± 26	56 ± 21
Glasgow coma score	10 ± 4	10 ± 5
OSF score	2.5 ± 1.0	1.9 ± 1.1

*Data are presented as No (%) or mean ± SD

**‡ **Criteria proposed by McCabe and Jackson

### Infections

Data concerning the infections are summarized in Table 2. Bacteremia, isolated or linked to another site of infection were more frequent in case of infections with PIP-TAZ R isolates (7/13 vs 9/70, p = 0.002). Other microorganisms were associated with *E. coli *in 30 cases. The germs more frequently isolated were *Pseudomonas aeruginosa *in 7 cases, *Klebsiella sp *in 5, *Streptococcus pneumoniae *in 5, and *Proteus mirabilis *in 4. Prior antibiotic prescription was reported in 8/13 (61%) infections with PIP-TAZ R isolates and in 29/70 (41%) infections with PIP-TAZ S isolates. Prior antibiotic regimen contained amoxicillin or amoxicillin/clavulanate in 8 cases (61%) of infections with PIP-TAZ R isolates and in 15 cases (21%) of infections with PIP-TAZ S isolates (p = 0.03). Time of onset of the infection from hospital admission was significantly longer in case of infections with PIP-TAZ R isolates as compared with PIP-TAZ S isolates (22 ± 32 vs 10 ± 21 days, p = 0.01).

**Table 2:** Infection characteristics and evolution during the ICU stay *.

**characteristics**	**PIP-TAZ R **n = 13	**PIP-TAZ S **n = 70	**p**
*Time of onset of the infection*			
From hospital admission	22 ± 32	10 ± 21	0.03
From ICU admission	7 ± 12	5 ± 10	NS
*Nosocomially/community acquired*	10 (77)	36 (51)	NS
*Locations of infection*			
Pneumonia	3 (27)	29 (41)	NS
Urinary tract	4 (31)	24 (34)	NS
Intra-abdominal	1 (9)	10 (14)	NS
Isolated bacteremia	4 (31)	1 (1)	0.007
Other sites	1 (9)	6 (9)	NS
*Infection-related complications*			
Septic shock	6 (46)	23 (33)	NS
ARDS	6 (46)	14 (20)	NS
MOF	4 (31)	14 (20)	NS
Superinfections	0	1 (1)	NS
Relapses	0	2 (3)	NS
*Outcome*			
Length of stay in ICU	26 ± 33	21 ± 22	NS
Death during ICU stay	6	27	NS

* Data are presented as No (%) or mean ± SD

### Outcome

The overall ICU mortality rate was 38% (expected hospital mortality according to SAPS2 was 61.9%). Of 13 patients with PIP-TAZ R *E. coli *infection, 6 (46%) died, compared with 27 (39%) of 70 patients with PIP-TAZ S *E. coli *infection (p = 0.7). The length of stay in ICU of the patients infected with PIP-TAZ R isolates was similar to the one of the patients infected with PIP-TAZ S isolates (26 ± 33 vs 21 ± 22 days, p = 0.7). The occurence of sepsis-related complications was similar wathever the resistance pattern for PIP-TAZ.

### Antibiotherapy

**Figure 1:**
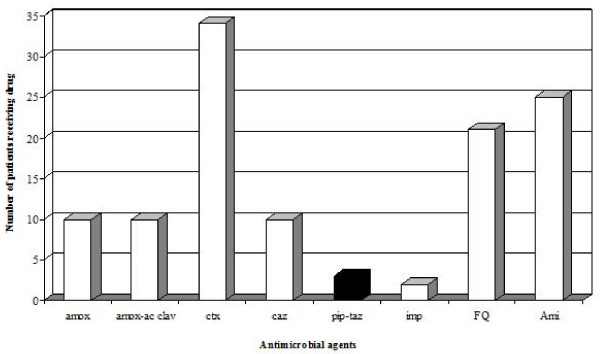
Initial antibiotherapy prescribed to patients infected with *Escherichia coli*. A patient could receive more than one drug.

**Figure 2:**
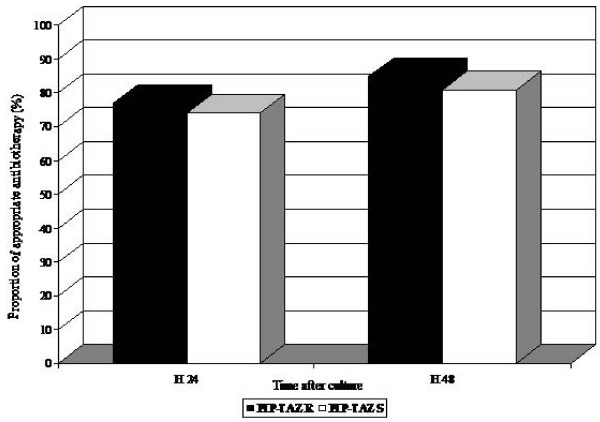
Percentages of patients infected with PIP-TAZ R isolates or with PIP-TAZ S isolates who were receiving appropriate antibiotic therapy (defined as at least 1 agent to which the infecting organism was susceptible) ≤ 24 h and ≤ 48 h after samples were obtained for culture.

Among the 83 patients, 77 received an antimicrobial therapy. Initial empiric treatment was a monotherapy in 2 cases (15%) of PIP-TAZ R *E. coli *infection, and in 37 cases (53%) of PIP-TAZ S *E. coli *infection (p = 0.02). The most commonly used empirical antibiotics were cefotaxime, amoxicillin alone or in combination with acid clavulanic, fluoroquinolones, and aminoglycosides (Figure 1). Aminoglycosides were always used in combination. Piperacillin/tazobactam was prescribed in only 3 cases. The proportion of patients receiving adequate antimicrobial therapy within 24 hours and 48 hours after diagnosis was similar whatever the resistance pattern of *E. coli *for PIP-TAZ (Figure 2).

## Discussion

In our retrospective cohort of ICU patients, 16% of the strains of *E. coli *isolated from clinically relevant specimens were resistant or intermediate to PIP-TAZ. This is far more than the rates observed by the French National Observatory for Epidemiology of Bacterial Resistance to Antimicrobials (ONERBA) that reports a 4% rate of PIP-TAZ intermediate or resistant strains among the strains of *E. coli *isolated in hospitalised patients [21]. In ICU, the PIP-TAZ resistance rate of *E. coli *is much higher. Our results confirmed those reported by Mohammedi et al. who noted an increase in the isolation rate of *E. coli *intermediate or resistant to PIP-TAZ in ICU patients from 6% in 1995 to 17% in 1999 [7]. But Mohammedi et al. did not characterise the mechanisms of resistance to PIP-TAZ. In our cohort, two among 13 PIP-TAZ resistant strains produced extended-spectrum β-lactamase. The production of high-level penicillinase was the predominant mechanism of resistance, identified in 11/13 PIP-TAZ resistant strains. This may have important consequences for the empirical choice of antimicrobial agents: these strains retained susceptibility to cefotaxime. The 2% (2/83) prevalence of ESBL strains in our cohort was similar to the prevalence recently described in French hospitals [22]. Recently, several studies revealed emergence of new ESBL-producing *E. coli *especially within the community, the CTX-M-producing isolates [23]. A limitation of our study is that underlying mechanisms of resistance were determined only using interpretative reading of the antibiogram. We did not performe any molecular study to identified the different β-lactamases.

In our cohort, a prolonged hospital stay prior to the diagnosis of *E. coli *infection or a recent antibiotic therapy containing amoxicillin or amoxicillin-acid clavulanic favoured infections with a PIP-TAZ resistant strain of *E. coli*. Mohammedi et al. conducted a multivariate analysis and identified prior use of amoxicillin and amoxicillin-acid clavulanic as a major risk factor of infection with PIP-TAZ resistant or intermediate strains of *E. coli *[8]. Their results were in accordance with previously published data establishing a link between prior antibiotherapy and infection with penicillin resistant strains [24,25]. In our study, as in any observational study, the missing data could have reduce the strength of our observations. The potential for unmeasured confounding data must be recognized.

In our cohort, we did not find any impact of the PIP-TAZ resistance phenotype on the prognosis of infections with *E. coli*. Several previous studies demonstrated that some patterns of resistance were significantly associated with mortality of *E. coli *infections [11,12]. Our study is the first analyzing the association of PIP-TAZ resistance with the outcome of *E. coli *infections. One explanation to the absence of prognosis impact of PIP-TAZ resistance in our cohort is that PIP-TAZ resistance did not result in a decrease of the proportion of adequate initial antimicrobial therapy. Monocentric nature of our study could explain this result, since PIP-TAZ was only rarely prescribed empirically in our ICU. In our cohort, predominance of medical admissions leading to a low proportion of intra-abdominal infections could have contributed to reduced prescription of PIP-TAZ. Urinary tract infections and pneumonia were the most frequent reported infections in our patients. PIP-TAZ is recommended for severe community-acquired intra-abdominal infections and for health care-associated intra-abdominal infections [4]. But, in case of pneumonia, it is recommended only for hospital-acquired pneumonia, ventilator-associated pneumonia, and health care-associated pneumonia in patients with late-onset disease or risk factors for multidrug-resistant pathogens [6]. Third generation cephalosporins are the reference treatment for complicated community-acquired urinary tract infections and is recommended for nosocomial urinary tract infections [26,27]. Another explanation to the absence of prognosis impact of PIP-TAZ resistance in our cohort is that our study was likely under powered to demonstrate a clinically important difference.

Our findings could have an impact on the choice of the empirical antibiotic prescription in case of Gram negative bacilli infection in critically ill patients. The selection of appropriate initial antibiotic therapy remains a challenge for the physician, who must balance the need for eradication of infection against the selection of resistant pathogens. The prognostic importance of appropriate initial antimicrobial therapy both in timing and efficacy on the causal micro-organism(s) has been underlined in many studies [28,29]. Prescription of PIP-TAZ in case of Gram negative bacilli infection in ICU is justified by its ability to treat both infections with enterobacteriaceae and with non-fermentative Gram-negative bacilli such as *Pseudomonas aeruginosa*, which are particularly frequent in ICU patients. The SOAP study conducted in European intensive care units revealed that the most common organisms isolated in the course of sepsis were *Staphylococcus aureus *(30%), *Pseudomonas species *(14%), and *Escherichia coli *(13%). The broad spectrum of activity of PIP-TAZ led some authors to consider it as the standard therapy for many infections such as ventilator-associated pneumonia and abdominal infections [30,31]. The high isolation rate of *E. coli *intermediate or resistant to PIP-TAZ that we report in ICU patients exposes to failure of empirical treatment with PIP-TAZ. Nevertheless our study does not allow to draw firm conclusions on the best empirical antibiotic therapy in case of *E. coli *infections in ICU. Finally, it highlights the importance of the local surveillance of the epidemiology of microorganisms in devising antibiotic strategies in a specific ICU.

## Conclusion

Our results indicate that infections with PIP-TAZ R *E. coli *are frequent in ICU patients. The PIP-TAZ resistance among *E. coli *strains is mainly due to production of high-level penicillinase. Prior use of amoxicillin may be a risk factor for PIP-TAZ R *E. coli *open to medical intervention. No prognostic impact of this pattern of resistance was found in our cohort. But, large prescription of PIP-TAZ for empirical initial antibiotic treatment of Gram negative bacilli infections in ICU may expose to inappropriate therapy and treatment failure.

## Competing interests

The authors declare that they have no competing interests.

## Authors' contributions

AM have contributed to acquisition of data, to analysis and interpretation of data, and to conception of the manuscript. JDR have contributed to conception and design of the study. GB and ME participated in acquisition and interpretation of data. GC have participated in interpretation of data. RM have contributed to analysis and interpretation of data. DD have conceived the study and contributed to interpretation of data.

## Pre-publication history

The pre-publication history for this paper can be accessed here:



## References

[B1] McDonald LC (2006). Trends in antimicrobial resistance in health care-associated pathogens and effect on treatment. Clin Infect Dis.

[B2] Vincent JL, Sakr Y, Sprung CL, Ranieri VM, Reinhart K, Gerlach H, Moreno R, Carlet J, Le Gall JR, Payen D (2006). Sepsis in European intensive care units: results of the SOAP study. Crit Care Med.

[B3] Quentin C, Arpin C, Dubois V, Andre C, Lagrange I, Fisher I, Brochet JP, Grobost F, Jullin J, Dutilh B (2004). Antibiotic resistance rates and phenotypes among isolates of Enterobacteriaceae in French extra-hospital practice. Eur J Clin Microbiol Infect Dis.

[B4] Solomkin JS, Mazuski JE, Baron EJ, Sawyer RG, Nathens AB, DiPiro JT, Buchman T, Dellinger EP, Jemigan J, Gorbach S (2003). Guidelines for the selection of anti-infective agents for complicated intra-abdominal infections. Clin Infect Dis.

[B5] Hughes WT, Amstrong D, Bodey GP, Bow EJ, Brown AE, Calandra T, Feld R, Pizzo PA, Rolston KV, Shenep JL (2002). 2002 guidelines for the use of antimicrobial agents in neutropenic patients with cancer. Clin Infect Dis.

[B6] American thoracic society (2005). Guidelines for the management of adults with hospital-acquired, ventilator-associated, and healthcare-associated pneumonia. Am J Respir Crit Care Med.

[B7] Mohammedi I, Tigaud S, Tournadre JP (2000). Emergence of piperacillin/tazobactam-resistant *Escherichia coli*. Intensive Care Med.

[B8] Mohammedi I, Ploin D, Duperret S, Chapuis F, Petit P (2003). Risk factors for piperacillin/tazobactam-resistant *Escherichia coli *in ICU patients: a clinical study. Intensive Care Med.

[B9] Vergis EN, Hayden MK, Chow JW, Snydman DR, Zervos MJ, Linden PK, Wagener MM, Schmitt B, Muder RR (2001). Determinants of vancomycin resistance and mortality rates in enterococcal bacteremia: a prospective multicenter study. Ann Intern Med.

[B10] Blot SI, Vanderwoude KH, Hoste EA, Colardyn FA (2002). Outcome and attribuable mortality in critically ill patients with bacteremia involving methicillin-susceptible and methicillin-resistant *Staphylococcus aureus*. Arch Intern Med.

[B11] Lautenbach E, Patel JB, Bilker WB, Edelstein PH, Fishman NO (2001). Extended-spectrum-β-lactamase-producing *Escherichia coli *and *Klebsiella pneumoniae*: risk factors for infection and impact of resistance on outcomes. Clin Infect Dis.

[B12] Lautenbach E, Metlay JP, Bilker WB, Edelstein PH, Fishman NO (2005). Association between fluoroquinolone resistance and mortality in *Escherichia coli *and *Klebsiella pneumoniae *infections: the role of inadequate empirical antimicrobial therapy. Clin Infect Dis.

[B13] Courvalin P, Goldstein F, Philippon A, Sirot J, Bruxelles MPC (1985). L'antibiogramme.

[B14] Comité de l'antibiogramme de la Société Française de Microbiologie (2007). Recommandations 2007. Paris: CASFM.

[B15] McCabe WR, Jackson CG (1962). Gram-negative bacteremia: etiology and ecology. Arch Intern Med.

[B16] Le Gall JR, Lemeshow S, Saulnier F (1993). Simplified Acute Physiologic Score. A new simplified acute physiologic score (SAPSII) based on European/North American multicenter study. JAMA.

[B17] Knaus WA, Draper EA, Wagner DP, Zimmerman JE (1985). Prognosis in acute organ system failure. Ann Surg.

[B18] Teasdale G, Jennet B (1974). Assessment of coma and impaired consciousness. Lancet.

[B19] Fagon JY, Chastre J, Novara A, Medioni P, Gibert C (1993). Characterization of intensive care unit patients using a model based on the presence or absence of organ dysfunctions and/or infection: the ODIN model. Intensive Care Med.

[B20] Bernard GR, Artigas A, Brigham KL, Carlet J, Falke K, Hudson L, Lamy M, Legall JR, Morris A, Spragg R (1994). The American – European consensus conference on ARDS: definition of mechanisms, relevant outcomes and clinical trial coordination. Am J Respir Crit Care Med.

[B21] Delarbre JM, Dubouix A, Robert J, Pour le Conseil Scientifique de l'ONERBA (2005). Résistance aux antibiotiques: des Chiffres de l'ONERBA au Bon Usage. Med Mal Inf.

[B22] Sirot J, Nicolas-Chanoine MH, Chardon H, Avril JL, Cattoen C, Croix JC, Dabernat H, Fosse T, Ghnassia JC, Lecaillon E (2002). Susceptibility of Enterobacteriaceae to β-lactam agents and fluoroquinolones: a 3-year survey in France. Clin Microbiol Infect.

[B23] Rodriguez-Bano J, Navarro MD, Romero L, Muniain MA, de Cueto M, Rios MJ, Hernandez JR, Pascual A (2006). Bacteremia due to ESBL producing *Escherichia coli *in the CTX-M era: a new clinical challenge. Clin Infect Dis.

[B24] Trouillet JL, Vuagnat A, Combes A, Kasss N, Chastre J, Gibert C (2002). *Pseudomonas aeruginosa *ventilator associated pneumonia: comparison of piperacillin resistant and piperacillin susceptible organisms. Clin Infect Dis.

[B25] Leflon-Guibout V, Ternat G, Heym B, Nicolas-Chanoine MH (2002). Exposure to co-amoxiclav as a risk factor for co-amoxiclav-resistant *Escherichia coli *urinary tract infection. J Antimicrob Chemother.

[B26] Wagenlehner FME, Naber KG (2006). Treatment of bacterial urinary tract infections: presence and future. European Urology.

[B27] Wagenlehner FME, Weidner W, Naber KG (2005). Emergence of antibiotic resistance amongst hospital-acquired urinary tract infections and pharmacokinetic/pharmacodynamic considerations. J Hosp Infect.

[B28] Iregui M, Ward S, Sherman G, Fraser VJ, Kollef MH (2002). Clinical importance of delays in the initiation of appropriate antibiotic treatment for ventilator-associated pneumonia. Chest.

[B29] Kang CI, Kim SH, Park WB, Lee KD, Kim HB, Kim EC, Oh MD, Choe KW (2005). Bloodstream infections caused by antibiotic-resistant Gram-negative bacilli: risk factors for mortality and impact of inappropriate initial antimicrobial therapy on outcome. Antimicrob Agents Chemother.

[B30] Fowler RA, Flavin KE, Barr J, Weinacker AB, Parsonnet J, Gould MK (2003). Variability in antibiotic prescribing patterns and outcomes in patients with clinically suspected ventilator-associated pneumonia. Chest.

[B31] Dupont H, Carbon C, Carlet J (2000). Monotherapy with a broad-spectrum beta-lactam is as effective as its combination with an aminoglycoside in treatment of severe generalized peritonitis: a multicenter randomized controlled trial. Antimicrob Agents Chemother.

